# Eco-friendly approach for spectrofluorimetric determination of moxifloxacin in pharmaceutical formulations and biological samples using deep eutectic solvents

**DOI:** 10.1186/s13065-026-01805-1

**Published:** 2026-04-18

**Authors:** Sarmad S. Muhammad, Wrya O. Karim, Naktal Al-Dulaimi

**Affiliations:** 1 Department of Chemistry, College of Science, Charmo University, Chamchamal- Sulaymaniyah, 46023 Kurdistan Region Iraq; 2 Department of Medical Laboratory Technology, Kalar Technical College, Garmian Polytechnic University, Kurdistan Region Kalar- Sulaymaniyah, 46021 Iraq; 3 Department of Chemistry, College of Science, University of Sulaimani, Sulaymaniyah, 46001 Kurdistan Region Iraq

**Keywords:** Moxifloxacin, Deep eutectic solvent, Ethaline, Spectrofluorimetry, Green chemistry, Pharmaceutical analysis

## Abstract

A novel green spectrofluorimetric technique was developed for the quantitative analysis of moxifloxacin (MOXI) in pure form, commercial formulations, and biological samples utilising deep eutectic solvent (DES) instead of an aqueous medium. Ethaline, a deep eutectic solvent composed of choline chloride and ethylene glycol, was utilised as the environmentally friendly solvent system. The approach exhibited enhanced analytical performance compared to water-based analysis, evidenced by a 1.2-fold increase in fluorescence intensity and a blue shift in both excitation and emission wavelengths. The calibration graph demonstrated a linear association between fluorescence intensity and moxifloxacin concentrations ranging from 0.2 to 10 µg mL^− 1^, with a correlation coefficient of 0.9993. The detection and quantification limits were established at 0.0165 µg mL^− 1^ and 0.0495 µg mL^− 1^, respectively. The influence of standard excipients was examined, although no interferences were identified. The established approach has been employed to study standard forms of moxifloxacin, commercial formulations, and biological samples. The approach, which effectively adhered to ICH standards, was employed for the examination of moxifloxacin in its pure state, pharmaceutical formulations, and biological specimens. The recovery percentages varied from 99.68% to 101.060% for pharmaceutical preparations and from 98.77% to 101.230% for human blood plasma and urine. This green analytical procedure can be used as a viable and green alternative for the routine pharmaceutical analysis with better sensitivity.

## Introduction

Fluoroquinolones represent some of the most potent antibacterial agents accessible for human use. These agents exhibit antibacterial efficacy against both Gram-positive and Gram-negative bacteria by inhibiting DNA gyrase, in addition to mycobacteria, mycoplasmas, and rickettsias [1].

Moxifloxacin (MOXI) is a fourth-generation 8-methoxy fluoroquinolone developed for the treatment of community-acquired pneumonia and upper respiratory tract infections. It targets Gram-negative pathogens, Gram-positive cocci, aerobic intracellular bacteria, atypical species, and anaerobic bacteria [2, 3].

The rising use of MOXI in clinical settings demands the creation of reliable, sensitive, and economical analytical techniques for its quantification in pharmaceutical formulations and biological matrices.

Numerous techniques for quantifying moxifloxacin have been recorded in pure concentrations and pharmaceutical formulations as well as biological fluids. These techniques, such as high-performance liquid chromatography (HPLC) [4–7], capillary electrophoresis [8], spectrophotometry [7, 9–10], and spectrofluormetric [11–14] techniques; however, the majority of these methods need organic solvents, complex sample preparation procedures, or necessitate costly instrumentation. The principle of green chemistry focus on design of chemical products and processes aimed at reducing or eliminating the use and generation of hazardous substances [15].

In recent years, there has been an increasing interest in the application of deep eutectic solvents (DESs) as environmentally sustainable solvents in analytical chemistry due to their eco-friendly characteristics and notable physicochemical qualities [16–18]. DESs are usually prepared by mixing a hydrogen bond acceptor (HBA) and a hydrogen bond donor (HBD), which interacts through strong hydrogen bonding to yield eutectic composition, whose melting point is much lower than each component. As a result, liquids with good solvent properties can form. DESs have several advantages over conventional organic solvents, such as low volatility, low toxicity, biodegradability, low cost, and simple preparation procedures without complicated purification steps [16–18]. As a result, DES has been extensively investigated in various analytical areas, such as extraction methods, sample preparation and chromatographic analysis [18, 19].

The spectrofluorimetry approach remains the most favoured method for assaying several types of pharmaceuticals in pure form, pharmaceutical formulations and biological samples due to its wide linearity dynamic range, relatively simple instrumentation, selectivity, high sensitivity, and notable economic benefits [20, 21]. Moxifloxacin, as a family of fluoroquinolones, is the best candidate for fluorometric analysis because it shows natural fluorescence due to its chemical structure [13, 22, 23]. The fluoroquinolone-DES interaction is capable of increasing fluorescence intensity due to changes in the surrounding environment as well as a reduction in non-radiative decay pathways [24, 25].

Although DESs are increasingly utilised in analytical chemistry, their potential to influence or enhance the fluorescence characteristics of analytes in spectrofluorimetric determinations remains inadequately explored.

This study presents the utilisation of choline chloride-ethylene glycol as a DES [26] as a fluorescence-enhancing medium for the quantification of MOXI in pharmaceutical formulations and biological samples. The aim of this study is to develop a simple, sensitive, and environmentally friendly spectrofluorimetric method for the quantitative analysis of MOXI utilising a DES solution as a green analytical medium.

## Materials and methods

### Chemicals and reagents

This study utilised analytical-grade reagents and chemicals. The standard moxifloxacin drug was provided by a pharmaceutical company, and commercial descriptions were acquired from the local pharmacies. MOXSOON^®^, DELMOXA^®^, and AVELOX^®^ each contain 400 mg of moxifloxacin per tablet, Choline chloride (ChCl) crystals (Sigma Aldrich, 98%).

ethylene glycol (EG) (Ajax Finechem, > 95%), distilled deionised water, glucose, fructose, lactose, sucrose, starch, Mg-stearate, and ascorbic acid were of analytical grade. Blood serum and urine samples were collected from healthy volunteers with informed consent.

### Preparation of DES

Ethaline DES was synthesised by combining hydrogen bond acceptors, choline chloride (HBAs), and hydrogen bond donors, ethylene glycol (HBDs), in specific molar ratios 1:2 within a 250 mL glass beaker. The mixtures were agitated and subsequently heated to 70 °C using a hot plate with continuous stirring for 1 h to achieve a homogenous and clear liquid. The prepared ethaline DES was permitted to cool to ambient temperature and store in a desiccator to prevent moisture absorption [26].

### Instrumentation

A Shimadzu RF-5301PC fluorescence spectrophotometer (Kyoto, Japan) was used for all the measurements, with excitation and emission slits at 5 mm, and 1 cm quartz cells.

### Preparation of moxifloxacin standard solutions

The moxifloxacin standard solution (100 µg mL^− 1^) was prepared by dissolving 0.005 g of the drug in 50 mL of ethaline DES and deionised water separately at room temperature. Standard working solutions with concentrations ranging from 0.2 to 10 µg mL⁻¹ were prepared by diluting appropriate aliquots of the stock solution with ethaline DES or deionised water as the diluent. The standard stock solution remained stable for a week under refrigeration.

### Sample solution preparation

Five tablets from each sample were weighed and then finely powdered in order to establish the average mass of the powder in one tablet. An accurately weighed quantity of powdered tablets (which is equivalent to 5 mg of MOXI content or approximately 8.66 mg of tablet powder) was dissolved in ethaline DES and transferred into a 50 mL volumetric flask and sonicated for about 15 min at ambient temperature (25 °C) until complete dissolution. The solution then was filtered through Whatman No. 1 filter paper to isolate the insoluble excipients. The transparent solution was diluted to the appropriate volume with ethaline DES to create a stock solution of 100 µg mL⁻¹ MOXI. Working concentrations within the linearity range of the proposed spectrofluorimetric technique were prepared.

### Biological sample preparation

To analyze biological samples, 1.0 mL of human blood plasma and urine were transferred separately into centrifuge tubes and spiked with an appropriate volume of 100 µg mL⁻¹ moxifloxacin standard solution. Protein precipitation was accomplished by adding 6.0 mL acetonitrile and vortexing.

The mixture was then centrifuged at 3000 rpm for 10 min to remove the precipitated proteins. The supernatant was transferred to a 50 mL volumetric flask and diluted to final concentrations of 0.2, 2, and 5 µg mL⁻¹ with ethaline DES for spectrofluorimetric analysis.

### Spectrofluorimetric measurements

The desired excitation and emission wavelengths of moxifloxacin were determined by recording the excitation and emission spectra of moxifloxacin in water and ethaline DES. The fluorescence intensity at the respective emission maxima was recorded for quantitative analysis of these bands upon excitation at an optimum wavelength. Fluorescence intensity versus MOXI concentrations was graphed to form calibration curves.

## Results and discussion

### Spectral characteristics and solvent effects

**Fig. 1:**
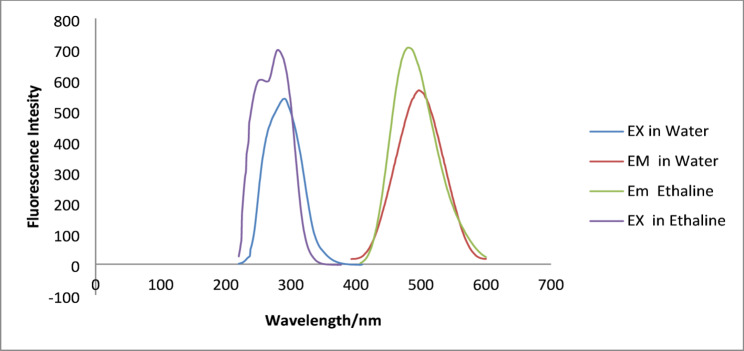
Excitation and Emission spectra of MOXI in Water and Ethaline

In order to assess the impact of the solvent microenvironment on the sperctroscopic property, the FL emission and excitation spectra of MOXI in both water and ethaline DES were displayed in Fig. 1. When moxifloxacin is dissolved in ethaline DES, its fluorescence behavior shows a notable blue shift in both excitation and emission wavelengths, as illustrated in Fig. 1. The maximum fluorescence intensity of MOXI in ethaline DES and water was seen at ʎ_exc_. = 280 nm and ʎ_em_. = 480 nm and at ʎ_exc_. = 290 nm and ʎ_em_. = 498 nm, respectively. The polarity and hydrogen bonding interactions in the ethaline DES medium differ from those in the aqueous solution, which explains this shifting [27, 28].

Furthermore, a 1.24-fold increase in fluorescence intensity has been noted for MOXI in ethaline DES as compared to water. This enhancement was explained by a number of factors: (1) the viscosity of ethaline DES limited non-radiative decay pathways, increasing fluorescence quantum yield [29]; (2) the unique hydrogen bonding network in DES stabilised the fluorophore’s excitation state [30, 31]; and (3) decreased solvent-induced quenching effects in the DES medium [32]. One of the main benefits of utilising DES as the solvent medium is the direct correlation between this fluorescence intensification and increased analytical sensitivity.

### Temperature effects

**Fig. 2:**
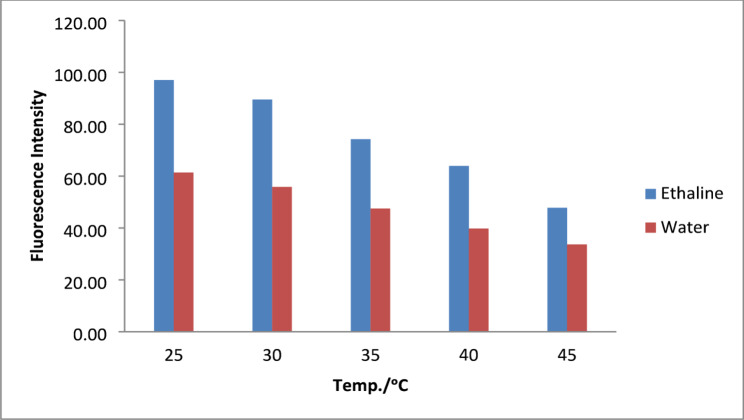
Effect of temperature on Fluorescence intensity of MOXI in Ethaline and Water

The moxifloxacin solution in both solvents was heated for 15 min at varying temperatures of 25, 30, 35, 40, and 45 degrees Celsius in an electrical thermostatic water bath to investigate the impact of increasing temperature on fluorescence intensity, as illustrated in Fig. 2. Although both DES and water showed temperature-dependent fluorescence quenching, ethaline DES consistently exhibited higher absolute fluorescence intensity across all temperatures in comparison to water. Elevated temperatures resulting in fluorescence quenching are probably correlated with a considerable increase of the non-radiative decay rates and molecular collisions [33]. Consequently, the analysis was performed at ambient temperature.

### Time effects (stability)

**Fig. 3:**
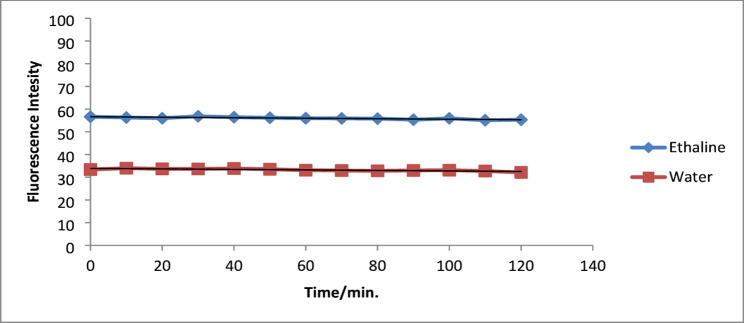
Effect of time on stability of Fluorescence intensity of MOXI in Ethaline and Water

The fluorescence stability of moxifloxacin was monitored for 120 min in ethaline DES and water at ambient temperature. The fluorescence intensity in both systems remained reasonably stable during the test period and gradually decreased over time, as illustrated in Fig. 3. In contrast, the ethaline DES systems demonstrated superior long-term stability, retaining over 97% of initial intensity within 120 min, compared to approximately 92% in water. The enhanced stability in DES results from the protective influence of the hydrogen bonding network, which mitigates photodegradation and oxidation [34].

### Method validation

The validity of the proposed approach was assessed utilizing the International Conference on Harmonization (ICH/Q2) methodology [35].

#### Linearity and sensitivity

**Fig. 4:**
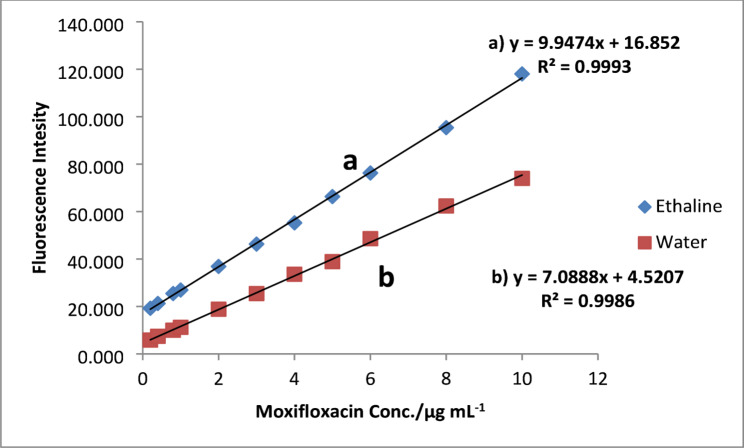
Linear range of MOXI. Conditions: 0.2–10 µg mL^− 1^ MOXI in; **a** Ethaline DES **b** Water

The calibration curve for fluorescence intensity against moxifloxacin concentration was established under optimal experimental conditions, Fig. 4 demonstrating linearity within the range of 0.2–10 µg mL^-1^ in both ethaline DES and water, with correlation coefficients of 0.9993 and 0.9986, respectively. The limit of detection (LOD) was determined using the equation LOD = 3.3 σ/S, where S is the slope of the calibration curve and σ is the standard deviation of the intercept. Table 1 displays the results of calculating different analytical parameters using the linear regression equation.

It is evident that the higher slope value for ethaline DES (9.9474 vs. 7.0888) attests to a higher fluorescence response and sensitivity of the DES assisted approach. The LOD and LOQ for ethaline DES were 0.0165 and 0.0495 µg mL^-1^, which are approximately 4-5-fold lower than the LOD (0.074 µg mL^-1^) and LOQ values (0.222 µg mL^-1^) in water. The method’s enhanced detection limits indicate that it can be used for trace analysis of MOXI in complex matrices. These low LOD and LOQ values are highly useful for the analysis of bioactive compounds when drug levels are expected to be quite low [36]. The suggested spectrofluorimetric method’s analytical performance was compared to previously reported MOXI determination methods, as summarized in Table 2. There are several different approaches for the analysis of MOXI in pharmaceutical formulations and biological fluids, including spectrofluorimetric, spectrophotometric, chromatographic and electrochemical methods [12, 13, 37–41]. Although these methods provide reasonably acceptable analytical results, some need expensive instrumentation and extensive sample preparation procedures, or they employ considerable amounts of organic solvents. On the other hand, the current approach uses a DES as an environmentally friendly solvent medium and offers a simple, rapid, and eco-friendly method for determining MOXI. This study used ethaline DES because of its comprehensive characterization of physicochemical parameters and its compatibility with fluorescence-based analytical instruments. Ethaline DES, characterized by its low toxicity, biodegradability, low volatility, and cost-effectiveness, aligns with the concepts of green analytical chemistry more effectively than other prevalent organic solvents [16–18, 30]. In addition, this work demonstrates that ethaline DES enhances MOXI fluorescence intensity and sensitivity relative to aqueous media. This enhancement results from the unique hydrogen-bonding network and microenvironmental polarity of the DES which inhibited non-radiative activities by stabilizing the excited state of moxifloxacin [30, 31]. Although DES systems often exhibit medium to high viscosity, this factor did not impact the performance of the technique. Conversely, the viscosity characteristic of ethaline DES significantly enhanced fluorescence intensity and stability by reducing non-radiative decay pathways as optimized by temperature and time studies [29]. Furthermore, the established approach exhibits a higher sensitivity than or is competitive with other previously published methods, with the advantage of utilising green solvent.

**Table 1:** Analytical parameters for MOXI assessment using spectrofluorimetry and calibration equations for MOXI in ethaline DES and aqueous solutions

Characteristics	Ethaline DES	Water
Wavelength of excitation (nm)	280	290
Wavelength of emission (nm)	480	498
Range of linearity, (µg mL^− 1^)	0.2–10	0.2–10
LOD, (µg mL^− 1^)	0.0165	0.0741
LOQ, (µg mL^− 1^)	0.0495	0.222
Standard deviation (SD) (µg mL^− 1^)	0.0667	0.1593
Regression formula	Y = 9.9474x + 16.852	Y = 7.0888x + 4.52
Slope	9.9474	7.0888
Intercept	16.852	4.52
Coefficient of correlation (r^2^)	0.9993	0.9986
RSD (%)	0.25	1.27

**Table 2:** Comparison of the proposed approach with other documented methods for determining MOXI levels

Technique	Linear range µg mL^− 1^	Medium/Solvent	LOD µg mL^− 1^	Reference
Spectrofluorimetry (micellar medium	0.03–0.30	Sodium dodecyl sulfate (SDS) in an acetate buffer pH of 4.	0.01	13
Spectrofluorimetry (Eu(III)-DOCA probe)	0.2-5	Ce(IV) in acidic medium	0.016	37
Spectrofluorimetry	0.2–10	Eosin Y in an acetate buffer pH of 3.6.	0.0322	38
UV-spectrophotometric	1–12	In 0.1 N hydrochloric acid (pH 1.2) and in phosphate buffer (pH 7.4	0.0402 and 0.0384	12
RP-HPLC	3-1300	Sodium dodecyl sulfate (SDS) in a phosphate buffer pH of 3.	1	39
Liquid chromatography	0.2-2	0.1% phosphoric acid	0.05	42
Electrochemical method	2–6	0.2 M phosphate buffer pH 3.2	0.175	41
Spectrofluorimetry-DES	0.2–10	Ethaline DES	0.0165	Current work

#### Selectivity and interference study

**Fig. 5:**
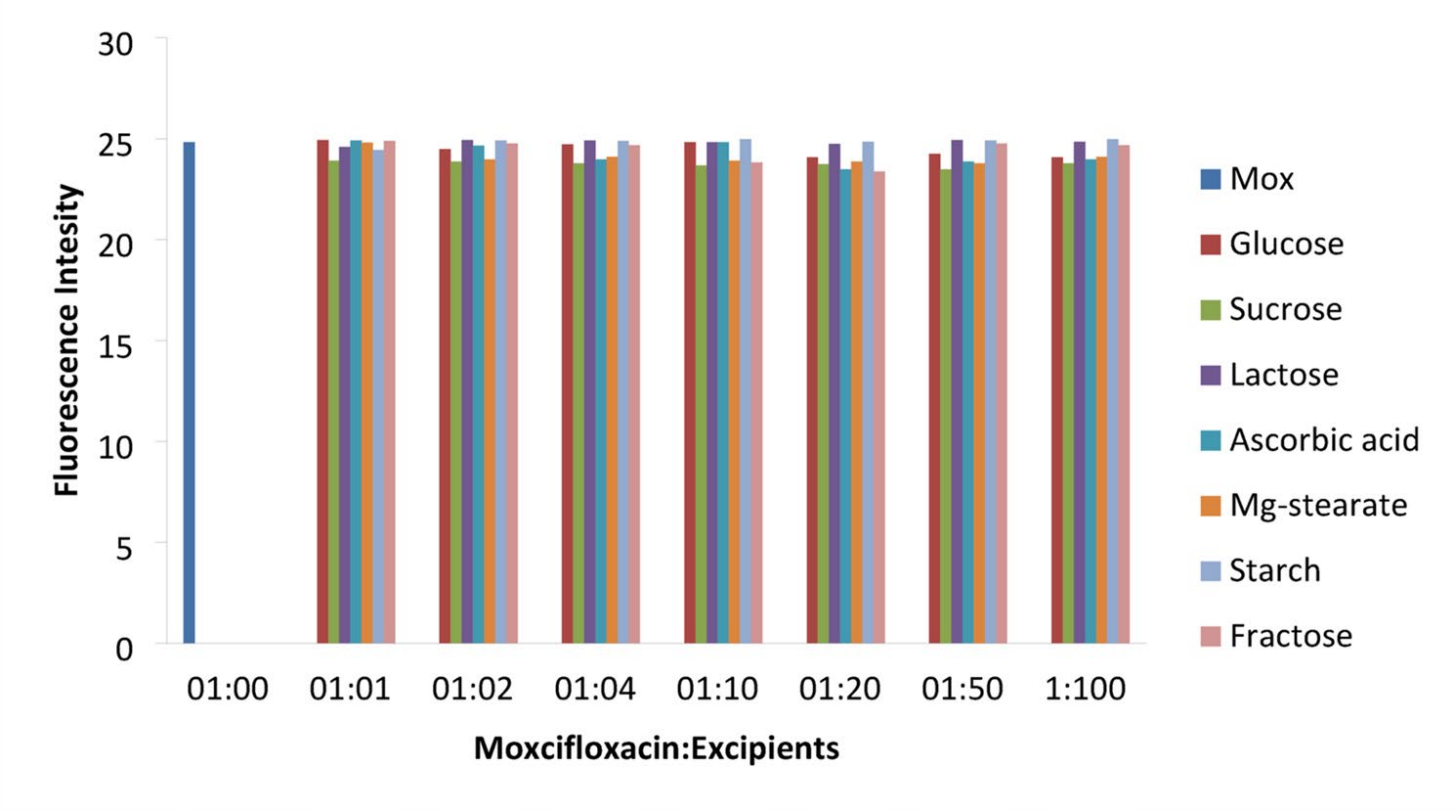
Effect of excipients on MOXI quantitation. Conditions; 0.8 µg mL^− 1^ MOXI in Ethaline DES

The selectivity of the method was assessed by analyzing the impact of tablet excipients such as glucose, sucrose, starch, sorbitol, lactose, and magnesium stearate using the standard addition technique. The proposed method involved the addition of these excipients at various concentrations in the following ratios: 1:1, 1:2, 1:4, 1:6, 1:8, 1:10, 1:20, 1:40, 1:50, and 1:100. None of the excipients exhibited significant interference in the determination of moxifloxacin, with recovery rates ranging from 95% to 101% (Fig. 5).

#### Specificity of the proposed method

The specificity of the proposed spectrofluorimetric approach was assessed by analyzing various sample types, including blank, placebo, standard moxifloxacin, and sample solutions.

The blank solution (ethaline DES) exhibited minimal fluorescence intensity at the emission wavelength of MOXI, so verifying the lack of background interference from the solvent system. A placebo solution comprising standard pharmaceutical excipients (such as lactose, starch, ascorbic acid, Mg-stearate…etc.) was analysed and demonstrated no significant fluorescence signal at the designated wavelength.

**Fig. 6:**
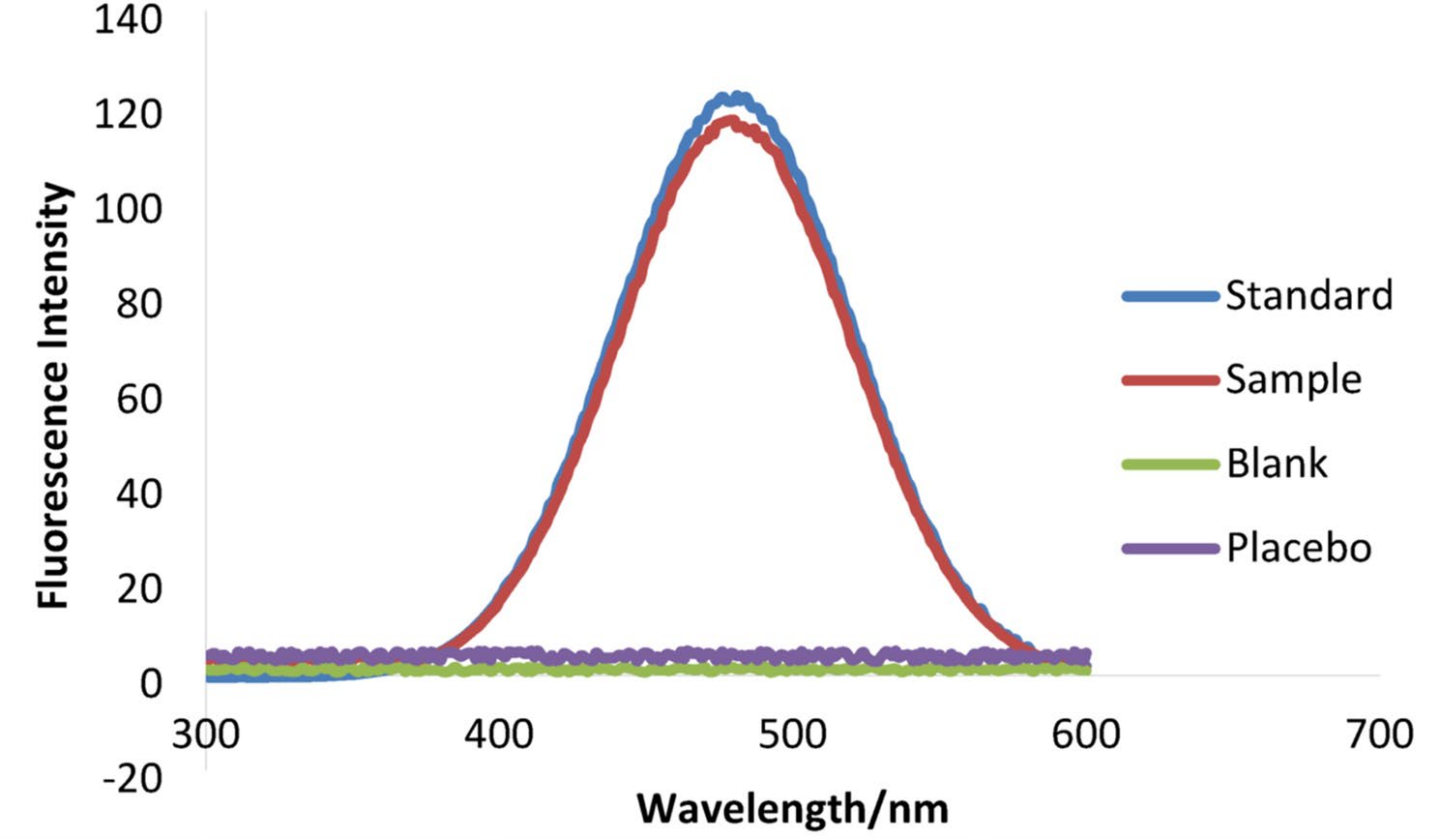
Emission fluorescence spectra of MOXI in: blank (ethaline), placebo, standard and sample solutions

In the opposite, for moxifloxacin in its standard solution and pharmaceutical sample solutions, highly defined and pronounced fluorescence peaks were observed. Figure 6 compares these spectra, showing no interference contribution from excipients and indicating the selectivity of the method.

#### Precision and accuracy

The precision of the method was confirmed by quantifying MOXI in both standard and commercial formulations, performed in triplicate at three different concentrations (0.2, 2.5, and 5 µg mL^-1^). Table 3 presents the results for the standard form, while Table 4 displays the outcomes for pharmaceutical formulations. The percent recovery achieved varied from 99.865% to 101.467% for the standard and from 98.363% to 102.50% for pharmaceutical formulations, exhibiting lower relative standard deviations and confirming the repeatability of the proposed method.

**Table 3:** The accuracy and precision of the current procedure of pure MOXI

Taken µg mL^-1^	Found µg mL^-1^	Recovery %	RSD%
0.2	0.203	101.467	0.2513
2.5	2.497	99.865	0.8908
5	5.009	100.153	0.2126

Mean = 100.495

SD = 0.195

RSD = 0.479

**Table 4:** The accuracy and precision of the current procedure of commercial MOXI

Pharmaceutical formulations	Taken µg mL^-1^	Found µg mL^-1^	Recovery %	RSD%
DELMOXA	0.2	0.198	99.021	0.494
	2.5	2.459	99.804	0.704
	5	5.027	100.550	1.347
MOXSOON	0.2	0.198	98.837	0.312
	2.5	2.459	98.363	0.732
	5	5.037	101.208	0.776
AVELOX	0.2	0.199	99.322	0.660
	2.5	2.482	99.266	2.038
	5	5.034	100.682	0.469

The results represent the medians of three distinct analyses.

The repeatability and reproducibility of the developed method were evaluated through the analysis of intra-day and inter-day precision. Intra-day precision was assessed by examining three distinct MOXI concentration levels (0.5, 2, and 8 µg mL^-1^) within the same day at six different times. Identical solutions were evaluated over three consecutive days to assess inter-day precision. Table 5 presents a summary of the findings.

**Table 5:** Intra-day and Inter-day precision of the proposed method

S	Concentration µg mL^-1^	*Intra-day precision* % Recovery ± RSD	*Inter-day precision* % Recovery ± RSD
1	0.5	99.202 ± 0.510	99.874 ± 0.442
2	2	101.139 ± 0.767	100.100 ± 1.204
3	8	100.313 ± 1.011	100.361 ± 0.929

The results represent the medians of three distinct analyses.

Lower RSD values indicate that the established method demonstrates both repeatability and reproducibility. This well performance can be explained based on the stable physicochemical properties of ethaline DES in terms of low volatility and a stable hydrogen bonding network, which will significantly reduce experimental errors [42].

The accuracy of the proposed method was evaluated through a standard addition approach involving three different brands of pills, each containing 400 mg of MOXI: DELMOXA, MOXSOON, and AVELOX. The known quantity of tablet solutions was combined with three different concentrations of standard MOXI solutions and analysed according to the specified procedure. The percentage recoveries varied between 98.50 and 101.16%, determined by comparing the findings prior to and following the addition of standard MOXI solution Table 6.

**Table 6:** Recovery percentage of MOXI in pharmaceutical formulations utilising the standard addition method

Pharmaceutical formulations	Amount added µg mL^-1^	Amount found µg mL^-1^	% Recovery ± RSD
DELMOXA	0.2	0.199	99.68 ± 1.52
	2.5	2.49	99.89 ± 0.31
	5	5.01	100.25 ± 1.13
MOXSOON	0.2	0.199	99.78 ± 0.43
	2.5	2.508	100.11 ± 0.29
	5	5.026	100.52 ± 1.08
AVELOX	0.2	0.199	99.88 ± 0.44
	2.5	2.5	100.01 ± 0.34
	5	5.05	101.06 ± 1.23

The results represent the medians of five distinct analyses.

## Application of the proposed method

### Determination of moxifloxacin in pharmaceutical formulations

**Fig. 7:**
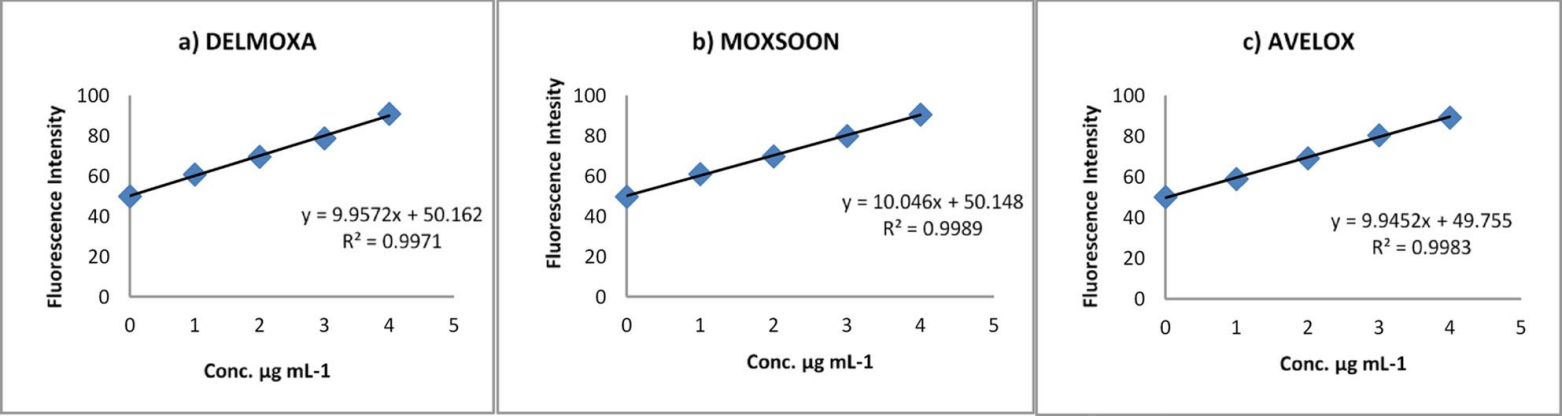
Standard addition calibration curves for determination of MOXI in: **a** DELMOXA **b** MOXSOON and **c** AVELOX tablet formulations

The proposed method was utilised to quantify MOXI in commercially available pharmaceutical formulations DELMOXA, MOXSOON, and AVELOX, and the findings are presented in Table 7. The contents that were labelled and those that were obtained using the proposed method were not significantly different. Recovery studies were conducted on the DELMOXA, MOXSOON, and AVELOX tablets (containing 400 mg of moxifloxacin) with the standard addition method. Figure 7 illustrates standard addition calibration curves for DELMOXA, MOXSOON, and AVELOX. The observed plots exhibited exceptional linearity, with excellent correlation coefficients, proving the lack of interference of the excipients in the determination of MOXI. Table 8 summarises the analytical parameters obtained from the standard addition calibration curves’ determination of MOXI in pharmaceutical formulations. The obtained recoveries ranged from 99.83% to 100.75%, validating the applicability of the proposed spectrofluorimetric technique.

**Table 7:** Examining the active components of MOXI utilised in pharmaceutical formulations

Pharmaceutical formulations	Active components Label value (mg/tablet)	Obtained value	% Recovery ± RSD
DELMOXA	400	403.02	100.75 ± 1.25
MOXSOON	400	399.35	99.83 ± 0.5
AVELOX	400	400.23	100.05 ± 0.86

The results represent the medians of five distinct analyses.

The findings from the interference investigation and standard addition approach support that the proposed technique is exceptionally specific for quantifying MOXI in the presence of potential interfering substances.

**Table 8:** Analytical parameters for MOXI assessment by standard addition calibration curves for determination of MOXI in pharmaceutical formulations

Pharmaceutical formulations	Regression equation	Slope	Intercept	*R* ^2^	%RSD
DELMOXA	y = 9.9572x + 50.162	9.9572	50.162	0.9971	1.25
MOXSOON	y = 10.046x + 50.148	10.046	50.148	0.9989	0.5
AVELOX	y = 9.9452x + 49.755	9.9452	49.755	0.9983	0.86

### Determination of moxifloxacin spiked serum and urine samples

The proposed method was employed to quantify MOXI in spiked human plasma and urine samples, owing to its high sensitivity and lack of interference from commonly utilised excipients. The findings are presented in Table 9. The recoveries of MOXI concentrations in urine and human plasma samples were 99.27–100.17% and 98.77–101%, respectively. Consequently, the proposed methodology can be utilised to quantify MOXI in serum and urine specimens with high precision.

**Table 9:** Determination of MOXI in spiked human plasma and urine samples

Sample	Spiked	Found	% Recovery ± RSD
Urine	0.2	0.199	99.27 ± 2.13
	2.5	2.500	100.17 ± 1.09
	5	4.998	99.98 ± 1.21
Human plasma	0.2	0.198	98.77 ± 0.49
	2.5	2.504	100.16 ± 1.09
	5	5.062	101.23 ± 1.51

## Conclusion

An ecofriendly spectrofluorimetric method has been developed and validated for the determination of moxifloxacin in ethaline DES as an analytical reagent. The developed method exhibits better analytical performance than that obtained by other aqueous systems, with a 1.2 times enhancement on the fluorescence; enhanced sensitivity (LOD = 0.0165 µg mL^-1^) and linearity (r² = 0.9993); and stable precision (RSD < 1%). The method was effectively used in the assay of MOXI in pharmaceutical dosage form and biological samples with recovery ranges between 98.77 and 101.23%, confirming its practical applicability.

The application of ethaline DES as an organic solvent alternative is in accordance with the principles of green analytical chemistry and provides significant environmental, safety and economic benefits. Feasible advantages of this method are simplicity, quickness, affordability and good analytical figures of merit, which allow its application as routine quality control analysis in pharmaceutical industries and bioanalytical laboratories.

Future work may involve developing different DES formulations to optimize analytical performance, application of the method for simultaneous determination of fluoroquinolones and construction of a portable fluorescence analyzer based on DES as sensors for point-of-care.

## Data Availability

The data used and analyzed during the current study are available from the corresponding author on reasonable request.
